# P-2100. Understanding barriers and facilitators for access to care in populations in Central California with Coccidioidomycosis (Valley fever) Meningitis using Consolidated Framework for Implementation Research!

**DOI:** 10.1093/ofid/ofaf695.2264

**Published:** 2026-01-11

**Authors:** Uday Chauhan, Rayne E Shepard, Nancy J Burke, Geetha Sivasubramanian

**Affiliations:** University of California San Francisco - Fresno, Fresno, CA; University of California, San Francisco - Fresno, Fresno, California; University of California, Merced, Merced, California; UCSF Fresno, Fresno, California

## Abstract

**Background:**

Long-term management of coccidioidal meningitis (CM) is challenging due to the need for lifelong antifungal therapy, frequent treatment failure, and barriers to follow-up among socioeconomically disadvantaged populations. These issues contribute to poor outcomes such as recurrent hospitalizations and shunt complications. This study aims to identify key barriers and facilitators to CM care in Central California using the Consolidated Framework for Implementation Research (CFIR), to inform future patient navigation interventions.

Consolidated Framework For Implementation Research (CFIR)

**Figure:**
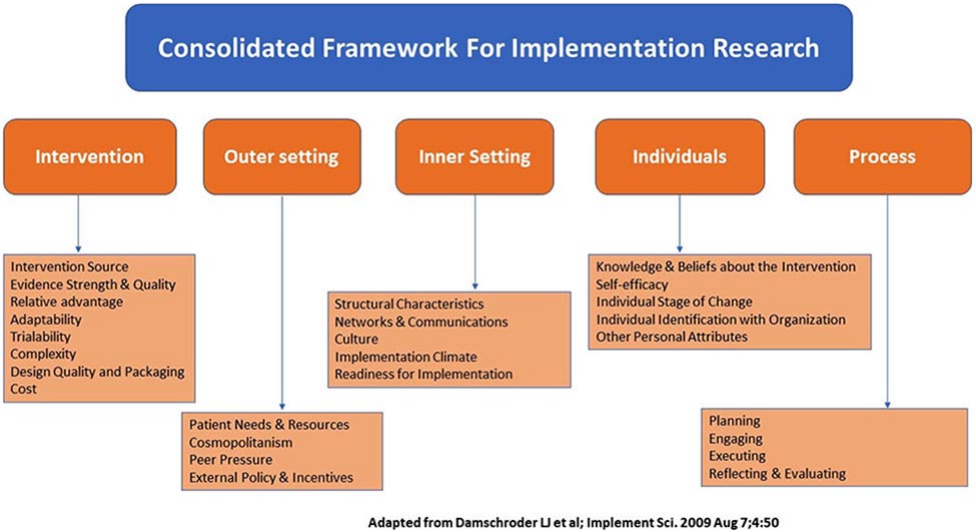

The diagram depicts the five interacting CFIR domains—Intervention, Outer Setting, Inner Setting, Individuals, and Process—with the constructs listed beneath each. Together, these multilevel factors shape the adoption, execution, and sustainability of evidence-based interventions. Adapted from Damschroder et al., Implementation Science 2009.

**Methods:**

Guided by CFIR (Figure 1), we launched a 3-phase mixed-methods study in California’s San Joaquin Valley. Phase 1 (in progress) involved semi-structured interviews with 7 CM patients (2013–2024) at two safety-net sites. Bilingual staff obtained oral consent and conducted interviews in person or by phone. Transcripts were coded to CFIR constructs to map barriers and facilitators. Phases 2–3 will include patient surveys (∼150) and provider/organizational assessments.

**Results:**

Among seven CM patients (median age 44 years; 4 males; 5 Hispanic), three CFIR-mapped themes emerged.

*Knowledge and communication gaps:* Six patients lacked understanding of “Valley Fever,” azole side effects, or lifelong therapy (e.g., “I only knew when the pills ran out”).

*Structural barriers:* Five reported ≥2-hour travel, 3–6-month waitlists, or interpreter shortages (“The shunt pain got worse while I waited months”).

*Financial/logistical burden:* Five faced high azole copays, insurance denials, or job loss; three had ≥3 shunt revisions.

Therapy was interrupted in four cases, adherence fell below 50% in three, and three were hospitalized within 12 months. Patients with active family advocates (4 of 7) navigated care more effectively, highlighting the potential of navigator models.

**Conclusion:**

Knowledge, structural, and financial barriers disrupt CM care, leading to therapy interruptions and rehospitalizations despite the availability of potent antifungals. CFIR-guided patient navigator interventions may help bridge these gaps; forthcoming patient surveys and provider assessments will inform a tailored implementation strategy for broader scale-up.

**Disclosures:**

All Authors: No reported disclosures

